# Comparison of transvaginal or transumbilical tissue extraction at laparoscopic gynecologic surgery: A 12‐year experience

**DOI:** 10.1002/ijgo.70050

**Published:** 2025-03-03

**Authors:** Osman Aşıcıoğlu, Berhan Besimoglu, Sinan Ateş

**Affiliations:** ^1^ Department of Obstetrics and Gynecology Trakya University Faculty of Medicine Edirne Turkey; ^2^ Department of Obstetrics and Gynecology Edirne Sultan I. Murat State Hospital Edirne Turkey

**Keywords:** adnexal mass, laparoscopic surgery, myomectomy, specimen extraction

## Abstract

**Objective:**

To present our surgical outcomes by comparing the transumbilical and transvaginal methods for the removal of specimens in laparoscopic surgery of fibroids and adnexal masses during our 12 years of experience.

**Methods:**

A retrospective cohort study was conducted at our referral center between January 2012 and April 2024. We evaluated surgical outcomes, patients' clinical‐demographic characteristics, cosmetic‐pain scores and dyspareunia by comparing the two methods that we use routinely.

**Results:**

We retrospectively reviewed 285 patients. Visual analog scale (VAS) scores at 24 h were lower in the transvaginal group than in the transumbilical group (0.4 ± 0.6 vs. 0.8 ± 0.8, *P* < 0.001). The 3‐month postoperative cosmetic score (CS) was higher in the transvaginal group than in the transumbilical group (4.5 ± 0.5 vs. 4.1 ± 0.6, *P* < 0.001). Furthermore, myomectomy and transumbilical were independent risk factors for lower VAS scores 24 h post surgery (myomectomy: odds ratio [OR] 3.42, *P* = 0.001, transvaginal route: OR 0.41, *P* = 0.005). Finally, the transumbilical extraction route and extension of the umbilical incision were independent risk factors for lower CS (*P* = 0.035 and *P* = 0.028).

**Conclusion:**

Removal of the specimen via the transvaginal route in laparoscopic adnexal mass and fibroid surgeries may lead to less pain in the early postoperative period and better cosmetic results without increasing the duration of the operation, the rate of intraoperative complications, and the rate of dyspareunia.

## 1 INTRODUCTION

Adnexal masses and uterine fibroids are frequent clinical situations on which gynecologists operate. For these operations, many gynecologists consider laparoscopy as the standard surgical technique because of its advantages, including better cosmetic results, early recovery time, and less postoperative pain.

In women of reproductive age, uterine fibroids are the most common uterine tumors,^1^, ^2^ and approximately one‐quarter of them require medical or surgical treatment.^3^ Besides fibroids, another important clinical issue for gynecologists is the management of adnexal masses. Depending upon the clinical features and nature of the adnexal mass, management can be either a conservative approach or a surgical intervention. The need for surgical intervention is 5.26%, in which 93% of adnexal masses originate from the ovary.^4^ Semm^5^ described the first laparoscopy technique for gynecologic surgery. An important aspect of laparoscopic gynecologic surgery is the removal of tissue from the abdominal cavity. The US Food and Drug Administration (FDA) has clearly stated that morcellation or dividing of tissue without an endo‐bag should be avoided in laparoscopic surgeries because of the fear of malignancy and malignant cells spreading into the abdominal cavity.^6^ For this reason, specimen extraction by morcellating within the bag is considered reasonable. The removal of fibroids or adnexal masses from the abdominal cavity can be by mini‐laparotomy incision, or lateral transabdominal, transumbilical, or transvaginal routes.

A mini‐laparotomy incision may increase postoperative pain, hernia, and wound infection rates. Furthermore, the expanded lateral transabdominal trocar incision may limit the cosmetic advantages of laparoscopy and increase the hernia rate.^7^, ^8^ As a result, the use of a transumbilical route without widening the extra port incision or the use of a natural orifice by posterior culdotomy appear to be the most appropriate methods for specimen removal. The vagina is an ideal natural orifice for tissue removal because of its flexibility and rich blood supply, allowing for quick and sequelae‐free healing. Many surgeons from different non‐gynecologic specialties have suggested the transvaginal route for the removal of surgical specimens.^9^ Chang et al.^10^ concluded that it is reasonable to use the transvaginal route to extract specimens without causing any sexual dysfunction or increasing local recurrence in patients with malignancy, but that more studies are required on this subject. In addition, although there are many studies on this subject in the field of gynecology, most of them evaluated only fibroids or adnexal masses. No study has evaluated both laparoscopic myomectomy and laparoscopic adnexal mass surgeries simultaneously.

In this study, we aimed to present our surgical outcomes by comparing transumbilical and transvaginal methods. These methods were routinely used for the removal of specimens in laparoscopic surgery of fibroids and adnexal masses during our 12 years of experience.

## 2 MATERIALS AND METHODS

**FIGURE 1:**
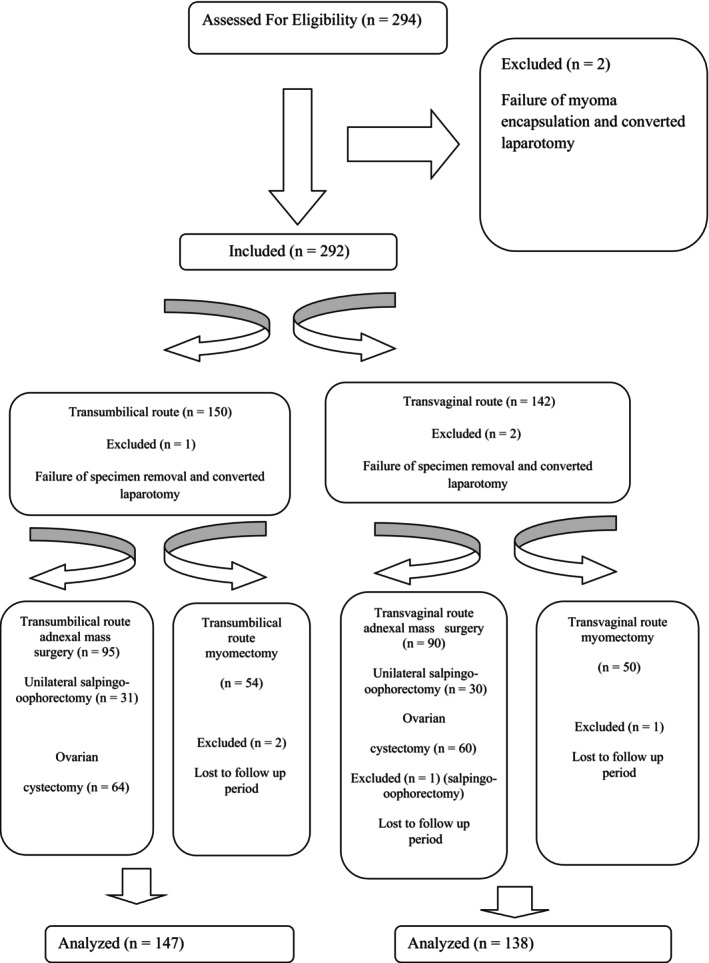
Flow diagram of research.

This retrospective cohort research was conducted at our referral center between January 2012 and April 2024. The flow diagram of the research is shown in Figure 1. The study was approved by the Trakya University Non‐Interventional Scientific Research Ethics Committee (TUTF‐GOBAEK 2024/389). All of the surgeries were implemented by the senior gynecologic endoscopic surgeon (OA), who is experienced in transumbilical or transvaginal extraction.

İnformed consent was obtained from all women. All women underwent preoperative transabdominal and transvaginal ultrasound examinations for the adnexal mass or fibroid, and morphology and size of masses were noted. Patients underwent myomectomy, cystectomy, or unilateral salpingo‐oophorectomy depending on the decision by the patient's clinic and surgeon. The route of specimen extraction was decided on by patient's choice by explaining the two methods routinely used by the surgeon.

Non‐inclusion criteria included: age younger than 18 or older than 75 years old, concomitant oncologic disease, suspected malignancy for adnexal mass, high anesthestic risk (American Society of Anesthesiologists score 3–4), fibroid size larger than 10 cm or fibroids in multiple locations that did not allow removal through laparoscopy, ongoing pregnancy, deeply infiltrating endometriosis, indications for concomitant total or subtotal hysterectomy, and in case of need for concomitant fibroid and adnexal mass surgery, bilateral adnexal mass surgery or another concomitant gynecologic surgery such as Burch colposuspension or cervical sacropexy.

We noted the body mass index (BMI; calculated as weight in kilograms divided by the square of height in meters; with obesity defined as a BMI over 30), postmenopausal status (defined retrospectively after 12 consecutive months of amenorrhea), parity, previous abdominal surgery, ovarian mass or fibroid dimensions, intraoperative and postoperative complications, the extra nonsteroidal analgesic requirement for the postoperative period, and postoperative incisional pain scores (assessed by a 10‐cm visual analog scale (VAS) at 1, 6 and 24 h). Preoperative hemoglobin levels were noted. Blood loss during surgery was evaluated by removing the washing fluid from the fluid in the graduated containers. Postoperative hemoglobin levels were measured, and the operation and specimen extraction times were calculated.

The operation time was described as the time from umbilical skin incision to closure. The specimen extraction time was described as the time from the incision in the posterior vaginal wall, through the extraction of the specimen, to the end of the colpotomy closure for the transvaginal group. For the transumbilical group, the time was described as the removal time of the specimen through the umbilical port site. This time was measured in minutes and recorded by the second assistant surgeon in the patient's file at the end of the operation. This time was measured in all laparoscopic operations where specimen removal was required.

The closed (Veress) technique was used and pneumoperitoneum was created using the Veress needle. Next, a 10‐mm trocar with a 30° operative laparoscope (Karl Storz) was introduced intraumbilically. Later, three additional 5‐mm trocars were inserted under direct visualization into the abdominal cavity. Two of them were at the levels of the lower abdominal quadrants (lateral to the medial umbilical ligament); another trocar was inserted from the middle of the lower left and umbilical trocar via a midline vertical skin incision. In general, the procedure was most commonly performed using a harmonic scalpel, bipolar electrocautery, and graspers. The adnexal masses were freed from their supports. In the presence of large cystic masses, puncture of the mass with unipolar electrocautery and controlled aspiration was conducted within a handmade glove bag. If intraoperative cyst rupture occurred, all fluid was suctioned out. For fibroid surgery, fibroids were freed from myometrial tissues.

**FIGURE 2:**
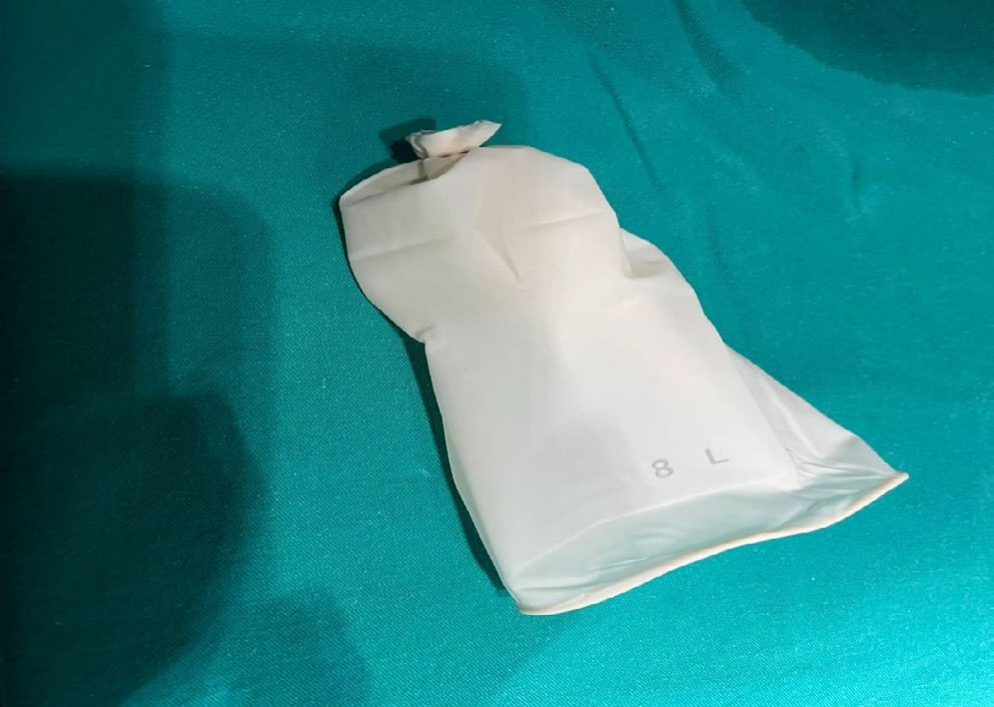
Glove bag.

Temporary bilateral uterine artery clips were routinely placed in laparoscopic myomectomies. The specimens were always extracted within a glove bag through a 10‐ to 12‐mm umbilical port incision or from the posterior fornix. We created a glove bag during the operation as follows: a sterile, surgical latex glove (size 8 or 8.5) was doubly tied at the level above the fingers, and then the fingers were removed. The glove was lubricated with sterile distilled water to remove the talcum powder and then introduced through the 10‐mm umbilical port (the optic port) (Figure 2). The specimen was placed into the glove bag under direct visualization. After that, one of two routes was selected. For the transumbilical route, the glove bag was pulled out through the umbilical incision together with the trocar. Once the orifice of the bag emerged from the incision, the umbilical port incision expanded to 2 cm (this last step, extension of the incision, was performed only in required cases). The specimen was aspirated or fragmented to ease removal; fibroids were extracted in fragments until the pieces were small enough to be removed through the umbilical incision.

For the transvaginal route, a posterior colpotomy was performed with a 2‐ to 3‐cm transverse incision using a harmonic scalpel, and the glove bag was removed through the vagina. If the specimen was too large for removal through the colpotomy incision, manual morcellation or dividing was performed within the glove bag. Then, under direct visualization, the specimen was extracted. After the procedure, the vagina was cleaned, and the colpotomy was closed transvaginally in a single layer using a 1–0 polyglactin suture. No rupture of a glove bag was identified for either of the two routes, and no local anesthetic agent was applied to the port sites.

Postoperative pain was managed with 75‐mg diclofenac sodium ampoules intramuscularly every 6 h for a maximum of four doses. Postoperative fluid maintenance (125 mL/h intravenously) was given for the first 24 h postoperatively. To reduce postoperative nausea and promote gastric protection, metoclopramide (20 mg) was given. The assistant doctor asked all patients to record the severity of incisional pain on a VAS 0 (no pain) and 10 (unbearable pain) at 1, 6 and 24 h after surgery. The VAS consists of a 10‐cm line, with two end points representing 0 (no pain) and 10 (unbearable pain). We routinely evaluate the VAS score of all patients who undergo laparoscopic surgery at our institution.

The patients were discharged on postoperative day one or two, and were advised to avoid sexual intercourse for 6 weeks following the surgery. All patients were checked at the outpatient clinic in the postoperative third month, and the presence of dyspareunia and cosmetic index scores were recorded. The cosmetic score (CS) is scored between 0 and 5 (0 being completely dissatisfied and 5 being extremely satisfied). The patient is asked to give a score between 0 and 5, and her statements are noted on the score card. If the cosmetic score is 3 or below, we use local anti‐inflammatory agents. If the patient is still not satisfied with the result, we consult a plastic and reconstructive surgery department. In dyspareunia, we apply a topical estrogen treatment.

The statistical analyses for this study were performed SPSS version 24.0 (IBM Corp., Armonk, NY, USA). The frequencies with percentages were used to define the categorical variables. All values are stated as means ± standard deviations (with a 95% confidence interval [CI]), unless additional information is stated. Continuous variables were compared using Student *t* test and the Mann–Whitney *U*‐test, whereas χ^2^ tests were used to compare the categorical data counts. Logistic regression analysis was performed to define the predictive factors. The results were introduced as odds ratios with 95% CI. In all analyses, a *P* value less than 0.05 indicated statistical significance.

## 3 RESULTS

In our cohort study, we retrospectively reviewed 285 patients. Of these, 101 underwent laparoscopic myomectomies, 60 underwent unilateral salpingo‐oophorectomy, and 124 received laparoscopic cystectomies (Figure 1). Table 1 shows the patient's clinical and demographic characteristics and surgical outcomes. All parameters were similar between the transumbilical and transvaginal groups. The rate of surgery types was similar between the groups (Table 1). The rate of additional anesthetic agent requirement was significantly higher in the transumbilical group (21.8% vs. 12.3%, *P* = 0.041), and all other parameters were similar between groups. Furthermore, during laparoscopic myomectomy, bladder injury occurred in one patient, and rectum injury occurred in another patient; both were repaired laparoscopically (Table 1). Additionally, the same parameters specifically for myomectomy and adnexal mass are shown separately in Tables 2 and 3.

**TABLE 1:** Clinical and demographic characteristics of patients and their operative outcomes.^a^

	Transvaginal (*n* = 138)	Transumbilical (*n* = 147)	*P* value
Age, years	35.3 ± 7.2	36.5 ± 6.6	0.143
Postmenopausal status	13 (9.4)	9 (6.1)	0.376
BMI	29.1 ± 3.5	30.0 ± 4.5	0.06
Obesity	58 (42.0)	69 (46.9)	0.474
Parity	1.1 ± 0.7	1.0 ± 0.9	0.588
Nulliparity	28 (20.3)	39 (26.5)	0.264
Myoma size, cm	8.2 ± 1.2	8.0 ± 2.0	0.773
Previous abdominal surgery	24 (17.4)	22 (15.0)	0.630
Extension of umbilical incision	0 (0)	30 (20.4)	
Surgery type			0.885
Unilateral salpingo‐oophorectomy	29 (21.0)	31 (21.1)	
Ovarian cystectomy	60 (43.5)	64 (43.5)	
Myomectomy	49 (35.5)	52 (35.4)	
Estimated blood loss, mL	37.2 ± 33.4	35.3 ± 33.9	0.878
Preoperative hemoglobin level, g/L	11.6 ± 1.4	11.4 ± 1.5	0.459
Postoperative hemoglobin level, g/L	10.3 ± 1.2	10.1 ± 1.1	0.986
Operative time, minutes	78.5 ± 15.0	76.4 ± 17.2	0.937
Extraction time, minutes	14.3 ± 5.8	13.8 ± 6.1	0.566
Intraoperative complication	1 (0.7)	1 (0.7)	
Postoperative complication	7 (5.1)	5 (3.4)	0.563
Postoperative fever	6 (4.3)	3 (2.0)	
Wound infection	1 (0.8)	2 (1.4)	
Additional analgesic requirements	17 (12.3)	32 (21.8)	0.041

Abbreviation: BMI, body mass index (calculated as weight in kilograms divided by the square of height in meters).

^a^ Data are presented as mean ± standard deviation or number (percentage).

**TABLE 2:** Laparoscopic adnexal mass surgery clinical characteristics and surgery outcome.^a^

	Transvaginal (*n* = 89)	Transumbilical (*n* = 95)	*P* value
Age, years	34.5 ± 7.6	36.1 ± 6.8	0.123
Postmenopausal status	9 (10.1)	6 (6.3)	0.424
BMI	29.4 ± 3.6	30.6 ± 4.5	0.165
Obesity	37 (41.6)	46 (48.4)	0.376
Parity	1.2 ± 0.6	1.1 ± 0.8	0.134
Nulliparity	13 (14.6)	25 (26.3)	0.068
Mass size, cm	8.6 ± 1.0	9.0 ± 1.7	0.070
Previous abdominal surgery	18 (20.2)	14 (14.7)	0.339
Extension of umbilical incision	0 (0)	9 (9.5)	
Unilateral salpingo‐oophorectomy	0 (0)	5 (5.3)	
Ovarian cystectomy	0 (0)	4 (4.2)	
Surgery type			
Unilateral salpingo‐oophorectomy	29 (32.6)	31 (32.6)	
Ovarian cystectomy	60 (67.4)	64 (67.4)	
Estimated blood loss, mL	12.9 ± 3.9	12.1 ± 2.9	0.112
Preoperative hemoglobin level, g/L	11.7 ± 1.4	11.5 ± 1.4	0.482
Postoperative hemoglobin level, g/L	10.2 ± 1.4	10.4 ± 1.1	0.640
Operative time, minutes	70.3 ± 7.5	68.4 ± 8.8	0.117
Extraction time, minutes	10.3 ± 1.8	9.8 ± 1.9	0.088
İntraoperative complication	0 (0)	0 (0)	
Postoperative complications	6 (6.7)	4 (4.2)	0.526
Postoperative fever	5 (5.6)	2 (2.1)	
Wound infection	1 (1.1)	2 (2.1)	
Additional analgesic requirements	9 (10.1)	16 (16.8)	0.203

Abbreviation: BMI, body mass index (calculated as weight in kilograms divided by the square of height in meters).

^a^ Data are presented as mean ± standard deviation or number (percentage).

**TABLE 3:** Laparoscopic myomectomy, clinical characteristics, and surgery outcome.^a^

	Transvaginal (*n* = 49)	Transumbilical (*n* = 52)	*P* value
Age, years	36.8 ± 6.1	37.2 ± 6.8	0.728
Postmenopausal status	4 (8.2)	3 (5.8)	0.710
BMI	28.9 ± 3.3	29.9 ± 4.5	0.200
Obesity	21 (42.9)	23 (44.2)	0.876
Parity	0.8 ± 0.8	1.0 ± 0.9	0.285
Nulliparity	15 (30.6)	13 (25.0)	0.657
Myoma size, cm	7.1 ± 0.9	6.6 ± 0.8	0.062
Previous abdominal surgery	6 (12.2)	8 (15.4)	0.648
Extension of umbilical incision	0 (0)	21 (40.4)	
Estimated blood loss, mL	81.0 ± 10.2	78.3 ± 11.8	0.112
Preoperative hemoglobin level, g/L	11.4 ± 1.5	11.3 ± 1.4	0.764
Postoperative hemoglobin level, g/L	10.3 ± 1.2	10.1 ± 1.2	0.527
Operative time, minutes	93.5 ± 13.6	96.7 ± 13.2	0.241
Extraction time, minutes	21.6 ± 2.6	20.9 ± 3.1	0.684
İntraoperative complication	1 (2.0)	1 (2.0)	
Postoperative complication	1 (2.0)	1 (2.0)	
Postoperative fever	1 (2.0)	0 (0)	
Wound infection	0 (0)	1 (2.0)	
Additional analgesic requirements	8 (16.3)	16 (30.8)	0.105

Abbreviation: BMI, body mass index (calculated as weight in kilograms divided by the square of height in meters).

^a^ Data are presented as mean ± standard deviation or number (percentage).

The VAS score at 1, 6 and 24 h post surgery, the CS, and the rate of dyspareunia at postoperative month 3 are compared in Table 4. VAS scores at 1, 6 and 24 h were lower in the transvaginal group than in the transumbilical group (*P* = 0.001, *P* < 0.001, *P* < 0.001). The third‐month postoperative CS was higher in the transvaginal group than in the transumbilical group (*P* < 0.001) (Table 4).

**TABLE 4:** Pain score, Cosmetic score, and dyspareunia.^a^

	Transvaginal (*n* = 138)	Transumbilical (*n* = 147)	*P* value
VAS, 1 h	5.0 ± 1.6	5.8 ± 2.0	0.001
VAS, 6 h	3.5 ± 1.1	4.0 ± 1.4	<0.001
VAS, 24 h	0.4 ± 0.6	0.8 ± 0.8	<0.001
Cosmetic score (3 months)	4.5 ± 0.5	4.1 ± 0.6	<0.001
Dyspareunia (3 months)	6 (4.3)	4 (2.7)	0.531

Abbreviation: VAS, visual analog scale.

^a^ Data are presented as reported as mean ± standard deviation or number (percentage).

**FIGURE 3:**
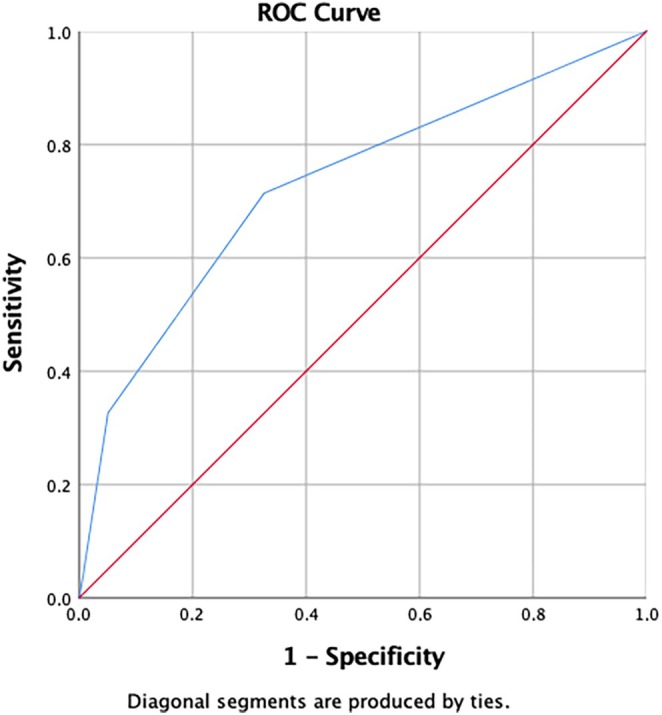
Cut‐off value for the visual analog scale at 24 h according to the area under the receiver‐operating characteristics (ROC) curve (AUC = 0.751).

**FIGURE 4:**
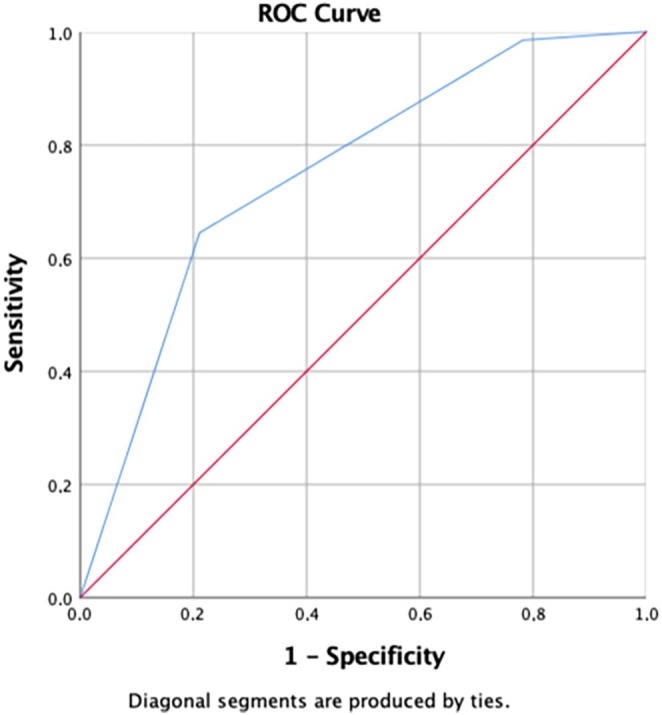
Cut‐off value for the cosmetic score at 3 months according to the area under the receiver‐operating characteristics (ROC) curve (AUC = 0.769).

When specimen extraction methods were used as state variables, the cut‐off value for the VAS score at 24 h post surgery was 3 (area under the curve [AUC]: 0.751; *P* = 0.001) and the cut‐off value for the CS at 3 months post surgery was 3 (AUC 0.769; *P* = 0.001) (Figures 3 and 4). Regression analyses were conducted for independent risk factors at a high VAS score at 24 h post surgery (≥3) and lower CS (≤3) as shown in Tables 4 and 5. Myomectomy and transumbilical routes were independent risk factors for lower VAS scores at 24 h (*P* = 0.001, *P* = 0.005, respectively) (Table 5). Finally, the transumbilical extraction route and extension of the umbilical incision were independent risk factors for lower CS (*P* = 0.035, *P* = 0.028, respectively) (Table 6).

**TABLE 5:** Multivariate analyses of risk factors for VAS 24‐h score of 3 or less.

	OR	95% CI	*P* value
Age (≥50 years)	0.61	0.11–3.62	0.258
Obesity	1.41	0.59–3.21	0.357
Nulliparity	1.19	0.58–2.51	0.512
Large mass or myoma (≥9 cm)	1.32	0.41–4.32	0.607
Previous abdominal surgery	1.82	0.92–3.71	0.082
Extension of umbilical incision	1.33	0.41–4.19	0.819
Myomectomy	3.42	1.72–6.90	0.001
Extraction route (transvaginal)	0.41	0.21–0.79	0.005

Abbreviations: CI, confidence interval; OR, odds ratio; VAS, visual analog scale.

**TABLE 6:** Multivariate analyses of risk factors for cosmetic scores of 3 or less (3 months).

	OR	95% CI	*P* value
Age (≥50 years)	0.51	0.11–2.62	0.458
Obesity	1.19	0.69–2.12	0.423
Nulliparity	1.71	1.01–2.89	0.474
Large mass or myoma size (≥9 cm)	0.52	0.19–1.02	0.069
Previous abdominal surgery	0.61	0.21–1.09	0.112
Extension of umbilical incision	4.13	2.01–8.29	0.028
Myomectomy	1.41	0.29–6.72	0.643
Extraction route (transvaginal)	0.21	0.11–0.40	0.035

Abbreviations: CI, confidence interval; OR, odds ratio.

## 4 DISCUSSION

This study showed that the transvaginal route for specimen extraction is better than the transumbilical route for acute incisional pain and cosmetic results. We routinely perform these two methods for specimen removal in laparoscopic surgery of adnexal masses and fibroids. Laparoscopic surgery is becoming increasingly important in fibroid and adnexal mass surgery, which is frequently performed in the gynecologic field. Nearly 6% of women with adnexal masses and 10% of women with fibroids will need surgery for treatment during their lifetime.^4^, ^11^, ^12^

The most important problem in laparoscopic surgery of fibroids and adnexal masses, especially in cases where hysterectomy is not performed concomitantly, is removing the large or solid specimen. Although fibroids are traditionally removed by direct electro‐morcellation within the abdomen, the US FDA and many other authors warn that fibroids should be removed as adnexal masses in a container bag to prevent the spread of possible malignant cells into the abdomen.^6^, ^7^, ^8^, ^9^, ^10^, ^11^, ^12^, ^13^ We routinely use glove bags instead of standard endo bags for specimen container bags because this method is considered both safe and cost‐effective.^14^, ^15^ In our study, as in previous articles, a glove bag was found to be effective and safe. During the study period, in the specimen extraction phase, no rupture was observed in any glove bag.

Given the concerns about morcellation without a bag, many methods have been described for the extraction of surgical specimens in laparoscopic myomectomy. In the early years, the authors used mini‐laparotomy for extraction.^16^, ^17^ In later years, it was reported that the mini‐laparotomy was associated with postoperative pain, infections, and undesirable cosmetic results. Later, lateral trocar incision enlargement was used for specimen extraction. Finally, many studies were published in which umbilical trocar incision area enlargement was described for specimen extraction. Expanding the transumbilical incision is one of the methods that we routinely use, even though there is no additional laterally extended 5‐mm trocar area incision in this method. Unlike lateral trocar incision enlargement, increasing the umbilical incision beyond 10 mm is associated with an increase in trocar area complications.^18^ Finally, the removal of surgical specimens through the vaginal route (natural orifice) was used by general surgeons and gynecologists, and better cosmetic results were reported.^19^, ^20^

Based on these results, gynecologists routinely prefer to use the vaginal route for the extraction of adnexal masses and fibroids. At first, Ghezzi et al.^21^ compared the transumbilical and transvaginal routes for laparoscopic adnexal mass extraction and reported that the transvaginal route was less associated with incisional pain. After this study, many other studies comparing these two methods for adnexal masses have been published, and the authors reported less incisional pain and better cosmetic results for the transvaginal route.^22^, ^23^, ^24^ Based on the results of our study, posterior culdotomy is a reasonable option for the removal of adnexal specimens in laparoscopic surgery.

Although transvaginal specimen extraction in laparoscopic myomectomies has been evaluated in numerous studies,^25^ no study comparing it with the transumbilical route exists in the literature. The difference and importance of this study is that there were laparoscopic myomectomy cases and adnexal mass cases in our study; the number of patients was higher than in many previous studies. In this study, we found better cosmetic and acute incisional pain results in the transvaginal route than the transumbilical route, without increasing the rate of dyspareunia. Therefore, we believe that the findings of our study are valuable and that the transvaginal route is more advantageous when added to laparoscopic myomectomies.

Among the most important findings of our research was the investigation of independent risk factors for pain scores and low CS. In addition, we believe that the presence of myomectomy cases, besides adnexal mass cases, increased the value of the findings. We determined that the transumbilical route and myomectomy are independent risk factors for a high VAS score (24 h post surgery). As a result, the finding that myomectomy is an independent risk factor is one of the most significant differences between our study and previous studies.^22^, ^23^ We also found that the transumbilical route and extension of the umbilical incision to 2 cm were independent risk factors for low CS, and that the need for extension of the umbilical incision to 2 cm was more often required in myomectomy cases. Therefore, although the transvaginal route is considered more suitable for both indications (fibroids and adnexal masses), its use in myomectomies appears to be more significant than adnexal mass surgery.

Another important finding of our study was that the duration of surgery in myomectomy cases, which was slightly longer compared with the literature, and the blood loss, was less.^25^ We attributed this result to our routine application of temporary bilateral uterine artery clips in all myomectomy cases. All other demographic characteristics and surgical results were compatible with the literature. Recently, Baradwan et al.^26^ argued that the temporary bilateral block of the uterine and utero‐ovarian arteries was an effective method for reducing surgical blood loss and the duration of hospital stay during laparoscopic myomectomy, similar to our study.

Finally, there may also be some limitations regarding our study. The retrospective design of our study and the absence of a 5‐mm camera for visualization during the transumbilical specimen removal can be considered limitations. In addition, the patient‐based decision on the route used to remove the specimen may have biased postoperative results. Also, being a single‐center instead of a multicenter study, and lacking the application of a scoring system such as the female sexual function scale, can be considered limitations of this study.

Despite these limitations, this research is a long‐term study that included laparoscopic myomectomy cases and adnexal mass cases. Furthermore, all the operations were conducted by the same senior surgeon. Finally, the similarity of demographic characteristics between the two groups and the absence of lost data due to an effective recording system strengthened the validity of our results.

In conclusion, removal of the specimen via the transvaginal route in laparoscopic adnexal mass and fibroid surgeries may lead to less pain in the early postoperative period and better cosmetic results without increasing the duration of the operation, the rate of intraoperative complications, and the rate of dyspareunia.

## AUTHOR CONTRIBUTIONS

All authors have accepted responsibility for the entire content of this manuscript and have approved its submission. OA contributed to the design, planning and conduct of this study, data acquisition and analysis, manuscript writing, and supervision. BB contributed to the critical revision of the manuscript and to data acquisition. SA contributed to data acquisition.

## CONFLICT OF INTEREST STATEMENT

The authors have no conflicts of interest.

## Data Availability

The authors declare that they have no conflicts of interest. The funding organization(s) played no role in the study design; in the collection, analysis and interpretation of data; in the writing of the report; or in the decision to submit the report for publication.
